# The Prevention of Consumption

**Published:** 1897-03

**Authors:** 


### The Prevention of Consumption.

Dr. B. W. Richardson, in *Asclepiad*, makes some suggestions which will prove beneficial to those having a tendency toward pulmonary tuberculosis. Dr. Richardson says that pure air for breathing is the first requisite for the prevention of consumption, and that a uniform climate and as much active out-door exercise as possible are essential. Out-door occupation is preventive. Amusements should favor muscular development and sustain healthy respiration. The dress of the consumptive should secure uniform warmth, and the hours of rest should be carefully regulated by the sunlight. Cleanliness, in the broadest sense of the word, is of special moment. The diet of consumptives should be ample, and every precaution should be taken to prevent colds.—*N. Y. Med. Record.*

